# Evaluation of antimicrobial susceptibility tests for *Acinetobacter* and *Pseudomonas* species using disks containing a high dose of meropenem

**DOI:** 10.1038/s41598-024-52538-x

**Published:** 2024-02-02

**Authors:** Shoichiro Endo, Tatsuya Tada, Satoshi Oshiro, Tomomi Hishinuma, Mari Tohya, Shin Watanabe, Jun-Ichiro Sekiguchi, Masaki Abe, Koji Nakada, Teruo Kirikae

**Affiliations:** 1 Department of Microbiology, Juntendo University School of Medicine, Tokyo, Japan; 2 Department of Clinical Laboratory, The Jikei University Daisan Hospital, Tokyo, Japan; 3 Department of Microbiome Research, Juntendo University School of Medicine, Tokyo, Japan; 4 Microbiology Research Division, Kohjin Bio Co., Ltd., Saitama, Japan; 5 AMR Research Laboratory, Juntendo Advanced Research Institute for Health Science, Juntendo University, Tokyo, Japan

**Keywords:** Microbiology, Medical research

## Abstract

The emergence and dissemination of carbapenem-resistant species of *Acinetobacter* and *Pseudomonas* have become a serious health concern. Routine antimicrobial disk susceptibility tests in clinical laboratories cannot distinguish between isolates that are highly carbapenem-resistant and those that are moderately carbapenem-resistant. The present study describes antimicrobial susceptibility tests using disks containing high doses (1000 μg) of meropenem. The diameters of inhibition zones were significantly negatively correlated with the MICs of *Pseudomonas* and *Acinetobacter* species for meropenem (R^2^: 0.93 and 0.91, respectively) and imipenem (R^2^: 0.75 and 0.84, respectively). Double disk synergy tests using clavulanic acid or sodium mercaptoacetate can detect ESBL or MBL producers. Susceptibility tests using disks containing high doses of meropenem can easily detect highly carbapenem-resistant isolates in a quantitative manner. These disks may be useful in bacteriological laboratories because of their technical ease, stability, and relatively low cost.

## Introduction

The emergence and spread of carbapenem-resistant species of *Pseudomonas* and *Acinetobacter* have become a serious health concern worldwide^1^. Infections with these microorganisms are associated with high mortality rates and limited treatment options^2^. Several mechanisms, including efflux pumps, porin channels and production of carbapenemases, contribute to carbapenem resistance in *Acinetobacter* and *Pseudomonas* species, with carbapenemases being especially responsible for high carbapenem resistance^3,4^. These bacteria frequently produce carbapenemases, including metallo-β-lactamases (MBLs), such as DIM-, IMP-, NDM- and VIM-type MBLs; as well as variants of OXA-type and extended-spectrum β-lactamases (ESBLs), such as GES-5, OXA-23, OXA-48 and OXA-72^3,4^. In bacteriological laboratories, however, routine antimicrobial susceptibility testing, including both disk diffusion and microdilution methods, are unable to quantify the levels of carbapenem resistance, because neither method has been adapted to analyze highly carbapenem-resistant isolates. Most carbapenemase-producing isolates of *Pseudomonas* and *Acinetobacter* species are highly resistant to carbapenems^4^, and several agents to infections caused by carbapenem-resistant isolates has been developed such as ceftazidime-avibactam, ceftolozane-tazobactam, meropenem-vaborbactam, imipenem-cilastatin-relebactam, plazomicin, eravacycline, and cefiderocol^5^. Therefore, it is clinically important to distinguish highly from moderately carbapenem-resistant isolates in clinical laboratories, which would also provide useful information about infection control practice in hospitals.

The mean peak plasma concentrations of carbapenems were actually 112 μg/ml (range: 83–140 μg/ml) after a 5-min intravenous bolus injection and 49 μg/ml (range 39–58 μg/ml) after a 30 min intravenous infusion, according to the product monograph^6^. Furthermore, different formula could be developed in infections with them, such as antimicrobial agents combined with carbapenemase-inhibitors and/or efflux pomp inhibitors^5^. Routine antimicrobial susceptibility testing of clinical isolates, including disk diffusion methods and twofold microdilution methods, cannot distinguish highly from moderately carbapenem-resistant isolates, because these methods have not been adapted to evaluation of highly carbapenem-resistant isolates^7,8^. The present study describes the development of antimicrobial susceptibility tests using disks containing high doses (1000 μg) of meropenem. This method was easily able to detect highly carbapenem-resistant *Pseudomonas* and *Acinetobacter* species, and it could be applicable especially in bacterial laboratories in developing countries where genetic diagnosis testing is not introduced.

## Methods

### Bacterial strains

All clinical isolates tested were obtained in the studies described in Tables 1 and 2, and their whole genome sequences had been determined using MiSeq (Illumina, San Diego, CA, USA) in the previous studies^9–26^. This study evaluated 125 clinical isolates of *Pseudomonas* species, comprising 111 isolates of *Pseudomonas aeruginosa*, 10 of *P. asiatica* and four of *P. monteilii*; and 112 clinical isolates of *Acinetobacter* species, comprising 105 isolates of *Acinetobacter baumannii*, three of *A. nosocomialis*, and one each of *A. bereziniae*, *A. indicus*, *A. pittii* and *A. radioresistens*. Of all the isolates tested, 84 *Pseudomonas* isolates and 85 *Acinetobacter* isolates were resistant to meropenem. All meropenem-resistant isolates of *Pseudomonas* species harbored genes encoding various types of β-lactamase and were expected to produce them, including various metallo-β-lactamases, including DIM-1-type (n = 1), both DIM-1- and NDM-1-type (n = 1), IMP-1-type (n = 5), IMP-1- and DIM-1-type (n = 1), IMP-6-type (n = 1), IMP-7-type (n = 1), IMP-43-type (n = 1), IMP-44-type (n = 1), NDM-1-type (n = 9), VIM-1-type (n = 8), VIM-2-type (n = 10), VIM-24-type (n = 6), and VIM-60-type (n = 3); and various GES-types of serine β-lactamases, including GES-1-type (n = 2), GES-5-type (n = 18), GES-6-type (n = 1), GES-15-type (n = 5), GES-24-type (n = 1), GES-26-type (n = 13), GES-30-type (n = 1) and KPC-2-type (n = 2) (All these β-lactamase-producing isolates were described in references cited in Table1.). Eighty meropenem-resistant isolates of *Acinetobacter* species harbored genes encoding various types of carbapenemase and were expected to produce them, including IMP-14 metallo-β-lactamase (n = 1); NDM-1-type metallo-β-lactamase (n = 4); NDM-1-type metallo-β-lactamase and OXA-type and PER-type serine β-lactamases (n = 4); NDM-1-type metallo-β-lactamase and OXA-type serine β-lactamase (n = 4); NDM-1-type metallo-β-lactamase and OXA-type, VEB-type and ADC (OXA-51-like) serine β-lactamases (n = 2); NDM-1-type metallo-β-lactamase and TEM-type and ADC serine β-lactamases (n = 1); and several types of serine β-lactamase, including OXA-, TEM-, PER-, VEB- and ADC-type serine β-lactamases (n = 71) (These isolates were described in references in Table 2.). *Acinetobacter baumannii* harbors an intrinsic *bla*_OXA-51-like_ gene encoding a charbapenemase, and an intrinsic *bla*_ADC_ encoding cephalosporinase, ADC (AmpC) β-lactamas, on chromosome; and IS*Aba1* flanked by the *bla*_OXA-51-like_ and *bla*_ADC_ are responsible for the production of OXA-51-like carbapenemase and the production of ADC cephalosporinase β-lactamase, respectively^2^. Two isolates of *A. baumannii* NCGM174 and NCGM211 listed in Table 2 were resistant to carbapenem and expected to produce ADC, because they harbored *bla*_ADC_ flanked by IS*Aba1*, but not other genes associated with known β-lactamases. *P. aeruginosa* ATCC27853 and PAO1 (ATCC15692) were obtained from the American Type Culture Collection (Manassas, VA, USA).

**Table 1:** *Pseudomonas* species isolates, carbapenemase produced by these isolates and their drug susceptibility profiles (MIC values and inhibition zone diameters using a high dose of meropenem disk).

Species	Strain	Carbapenemase	MICs (μg/mL)				Inhibition zone diameter (mm)	References
			IPM	MEM	AMK	CIP		
*P. aeruginosa*	PAO1 (ATCC15692)	–	2	2	8	0.5	50	–
*P. aeruginosa*	ATCC 27853	–	2	2	2	0.25	50	–
*P. aeruginosa*	09181	–	1	2	8	0.25	58	This study
*P. aeruginosa*	083141	–	1	2	16	0.5	49	This study
*P. aeruginosa*	091618	–	1	2	16	0.5	49	This study
*P. aeruginosa*	091743	–	1	2	8	0.25	51	This study
*P. aeruginosa*	091813	–	1	2	8	0.25	49	This study
*P. aeruginosa*	091818	–	1	2	8	0.25	49	This study
*P. aeruginosa*	092737	–	2	2	8	0.25	50	This study
*P. aeruginosa*	093020	–	2	2	8	0.25	55	This study
*P. aeruginosa*	100118	–	1	2	16	0.5	48	This study
*P. aeruginosa*	100211	–	1	2	8	0.25	50	This study
*P. aeruginosa*	IMCJ2. S1	IMP-1	64	512	128	32	15	^16^
*P. aeruginosa*	IOMTU 133	DIM-1	16	64	2048	32	35	^13^
*P. aeruginosa*	IOMTU 9	NDM-1	512	1024	2048	64	21	^13^
*P. aeruginosa*	JUNP 146	KPC-2	256	256	32	8	26	^19^
*P. aeruginosa*	JUNP 536	KPC-2	512	512	32	32	18	^19^
*P. aeruginosa*	JUPA 4001	VIM-24	1024	1024	32	8	16	^18^
*P. aeruginosa*	MyNCGM 456	NDM-1	256	256	128	128	25	^11^
*P. aeruginosa*	MyNCGM 514	DIM-1, NDM-1	256	512	128	128	27	^11^
*P. aeruginosa*	MyNCGM145	IMP-1	32	64	128	32	26	^11^
*P. aeruginosa*	MyNCGM198	IMP-1	8	64	128	32	30	^11^
*P. aeruginosa*	MyNCGM202_3	IMP-1, DIM-1	256	128	64	128	29	^11^
*P. aeruginosa*	MyNCGM219	IMP-1	8	128	64	32	27	^11^
*P. aeruginosa*	MyNCGM314	IMP-1	16	64	64	32	29	^11^
*P. aeruginosa*	NCGM 1438	IMP-7	64	128	32	8	23	^20^
*P. aeruginosa*	NCGM 1496	IMP-43	512	512	16	16	9	^14^
*P. aeruginosa*	NCGM 1663	IMP-44	512	4096	32	32	6	^14^
*P. aeruginosa*	NCGM 1890	GES-5	64	128	16	32	21	^12^
*P. aeruginosa*	NCGM 1957	GES-5	64	128	64	32	21	^12^
*P. aeruginosa*	NCGM 2100	GES-5	64	64	128	32	31	^12^
*P. aeruginosa*	NCGM 2677	GES-5	64	128	32	32	24	^12^
*P. aeruginosa*	NCGM 2727	VIM-1	512	512	8	8	29	^18^
*P. aeruginosa*	NCGM 2842	GES-5	32	64	32	64	25	^12^
*P. aeruginosa*	NCGM 2854	GES-1	16	4	16	16	40	^12^
*P. aeruginosa*	NCGM 2858	GES-5	64	128	32	32	25	^12^
*P. aeruginosa*	NCGM 2860	GES-5	32	128	64	8	29	^12^
*P. aeruginosa*	NCGM 2879	GES-5	64	128	64	8	25	^12^
*P. aeruginosa*	NCGM 2883	GES-5	32	128	32	16	26	^12^
*P. aeruginosa*	NCGM 2893	VIM-1	512	256	16	8	27	^18^
*P. aeruginosa*	NCGM 2894	GES-5	32	128	16	8	28	^12^
*P. aeruginosa*	NCGM 2900	GES-5	32	128	32	16	27	^12^
*P. aeruginosa*	NCGM 2903	GES-5	32	128	32	16	26	^12^
*P. aeruginosa*	NCGM 2909	GES-5	32	64	32	8	29	^12^
*P. aeruginosa*	NCGM 2910	VIM-1	512	256	16	8	17	^18^
*P. aeruginosa*	NCGM 2928	GES-5	64	128	64	8	27	^12^
*P. aeruginosa*	NCGM 2940	GES-5	64	128	64	16	27	^12^
*P. aeruginosa*	NCGM 2946	GES-5	64	512	64	16	21	^12^
*P. aeruginosa*	NCGM 2952	GES-5	64	128	32	32	25	^12^
*P. aeruginosa*	NCGM 2957	GES-5	64	128	32	32	25	^12^
*P. aeruginosa*	NCGM 3037	VIM-1	256	256	4	4	26	^15^
*P. aeruginosa*	NCGM 3046	VIM-1	512	512	4	8	25	^18^
*P. aeruginosa*	NCGM 3105–2	GES-30	16	64	32	32	31	^12^
*P. aeruginosa*	NCGM 3164	VIM-2	256	64	64	16	30	^15^
*P. aeruginosa*	NCGM 3188	IMP-43	1024	2048	32	16	6	^14^
*P. aeruginosa*	NCGM 3197	IMP-6	32	512	32	8	12	^15^
*P. aeruginosa*	NCGM 3209	GES-1	8	8	32	16	34	^12^
*P. aeruginosa*	NCGM 3240	VIM-1	512	256	16	16	21	^18^
*P. aeruginosa*	NCGM 3264	VIM-1	512	512	4	4	23	^18^
*P. aeruginosa*	NCGM 3316	GES-24	32	128	512	256	30	^12^
*P. aeruginosa*	NCGM 3338	GES-26	16	8	32	16	39	^12^
*P. aeruginosa*	NCGM 3352	GES-26	8	8	16	16	39	^12^
*P. aeruginosa*	NCGM 3357	GES-26	16	4	16	16	37	^12^
*P. aeruginosa*	NCGM 3376	GES-26	8	8	16	16	38	^12^
*P. aeruginosa*	NCGM 3388	GES-6	64	128	64	16	19	^12^
*P. aeruginosa*	NCGM 3416	GES-26	8	4	16	8	38	^12^
*P. aeruginosa*	NCGM 3422	GES-26	8	4	8	16	39	^12^
*P. aeruginosa*	NCGM 3424	VIM-1	512	256	8	32	30	^18^
*P. aeruginosa*	NCGM 3447	GES-26	16	8	16	16	39	^12^
*P. aeruginosa*	NCGM 3449	VIM-60	1024	2048	64	16	19	^18^
*P. aeruginosa*	NCGM 3454	GES-26	16	8	16	16	37	^12^
*P. aeruginosa*	NCGM 3459	GES-26	8	4	16	16	39	^12^
*P. aeruginosa*	NCGM 3468	GES-15	16	16	32	32	33	^12^
*P. aeruginosa*	NCGM 3472	GES-26	16	4	16	16	40	^12^
*P. aeruginosa*	NCGM 3486	GES-26	1024	512	64	32	20	^12^
*P. aeruginosa*	NCGM 3508	GES-15	8	8	16	32	39	^12^
*P. aeruginosa*	NCGM 3517	VIM-60	1024	2048	64	32	11	^18^
*P. aeruginosa*	NCGM 3521	GES-15	8	8	16	32	37	^12^
*P. aeruginosa*	NCGM 3538	GES-15	8	4	16	32	39	^12^
*P. aeruginosa*	NCGM 3546	GES-15	16	16	16	16	39	^12^
*P. aeruginosa*	NCGM 3563	GES-26	16	8	16	16	41	^12^
*P. aeruginosa*	NCGM 3573	GES-26	16	8	32	16	37	^12^
*P. aeruginosa*	NCGM 3596	VIM-24	256	512	32	32	12	^18^
*P. aeruginosa*	NCGM 3741	VIM-24	256	512	16	16	13	^18^
*P. aeruginosa*	NCGM 3750	VIM-60	512	2048	16	16	14	^17^
*P. aeruginosa*	NCGM 3798	VIM-2	512	512	128	16	24	^18^
*P. aeruginosa*	NCGM 3799	VIM-24	512	4096	16	32	8	^18^
*P. aeruginosa*	NCGM 3814_2	VIM-24	512	256	8	64	18	^18^
*P. aeruginosa*	NCGM 3822	VIM-24	256	512	16	16	17	^18^
*P. asiatica*	My 34	NDM-1	32	1024	4096	1024	14	^10^
*P. asiatica*	My 371	NDM-1	512	4096	64	512	9	^10^
*P. asiatica*	My 545	NDM-1	256	4096	128	512	9	^10^
*P. asiatica*	My 569	NDM-1	256	4096	16	64	13	^10^
*P. asiatica*	My 601–1	NDM-1	512	4096	16	512	12	^10^
*P. asiatica*	My 660	VIM-2	64	64	32	256	29	^10^
*P. asiatica*	My 680	NDM-1	512	2048	32	1024	6	^10^
*P. asiatica*	My 756	NDM-1	256	1024	256	256	14	^10^
*P. asiatica*	Ryu 5	VIM-2	16	32	4	0.5	36	^9^
*P. asiatica*	Ryu 7	VIM-2	32	32	4	0.5	34	^9^
*P. monteilii*	RYU 165	VIM-2	256	256	16	64	26	^9^
*P. monteilii*	Ryu 6	VIM-2	128	128	8	32	30	^9^
*P. monteilii*	Ryu 8	VIM-2	256	256	16	32	30	^9^
*P. monteilii*	Ryu 9	VIM-2	64	128	16	32	29	^9^
*P. aeruginosa*	JU001	–	2	0.0625	4	0.5	61	This study
*P. aeruginosa*	JU002	–	2	0.5	4	0.125	58	This study
*P. aeruginosa*	JU003	–	4	0.25	8	0.125	59	This study
*P. aeruginosa*	JU004	–	4	0.25	8	0.125	60	This study
*P. aeruginosa*	JU005	–	2	0.125	8	0.125	59	This study
*P. aeruginosa*	JU006	–	2	4	4	0.125	37	This study
*P. aeruginosa*	JU007	–	4	025	4	0.125	58	This study
*P. aeruginosa*	JU008	–	4	0.0625	8	1	60	This study
*P. aeruginosa*	JU009	–	2	0.25	4	0.125	59	This study
*P. aeruginosa*	JU010	–	1	0.25	2	0.125	60	This study
*P. aeruginosa*	JU011	–	4	0.625	4	1	61	This study
*P. aeruginosa*	JU012	–	4	1	4	0.125	58	This study
*P. aeruginosa*	JU013	–	4	2	4	0.125	49	This study
*P. aeruginosa*	JU014	–	4	1	4	0125	59	This study
*P. aeruginosa*	JU015	–	1	2	8	1	51	This study
*P. aeruginosa*	JU016	–	4	0.125	4	0.125	60	This study
*P. aeruginosa*	JU017	–	16	0.125	2	0.0625	59	This study
*P. aeruginosa*	JU018	–	4	0.5	4	0.125	60	This study
*P. aeruginosa*	JU019	–	8	2	4	0.125	49	This study
*P. aeruginosa*	JU020	–	4	0.5	8	0.125	59	This study
*P. aeruginosa*	JU024		8	0.125	0.5	0.0625	59	This study
*P. aeruginosa*	JU025		1	0.5	128	32	59	This study
*P. aeruginosa*	JU034		4	0.5	4	0.0625	42	This study

**Table 2:** *Acinetobacter* species isolates, carbapenemases produced by these isolates and their drug susceptibility profiles (MIC values and inhibition zone diameters using a high dose of meropenem disk).

Species	Strain	Carbapenemase	MICs (μg/mL)				Inhibition zone diameter (mm)	References
			IPM	MEM	AMK	CIP		
*A. baumannii*	120939	–	2	2	2	0.25	36	This study
*A. baumannii*	121101	–	2	2	2	0.25	40	This study
*A. baumannii*	122226	–	2	2	2	0.25	38	This study
*A. baumannii*	MyNCGM 134_2	OXA-23, OXA-66	32	32	4096	128	27	^15^
*A. baumannii*	MyNCGM 135_2	OXA-23, OXA-64	16	32	256	128	25	^15^
*A. baumannii*	MyNCGM 140	OXA-23, OXA-58, OXA-66	8	32	512	32	27	^15^
*A. baumannii*	MyNCGM 158	OXA-23, OXA-58	8	16	64	16	32	^15^
*A. baumannii*	MyNCGM 17	OXA-23, OXA-66	16	16	4096	64	30	^15^
*A. baumannii*	MyNCGM 320	OXA-23like, OXA-91	32	64	4096	256	23	^15^
*A. baumannii*	MyNCGM 370	OXA-69 (OXA-51-like^a^)	32	64	4096	16	24	^15^
*A. baumannii*	MyNCGM 58	OXA-23, OXA-51-like	32	16	2	128	33	^15^
*A. baumannii*	MyNCGM 60	OXA-23, OXA-66	32	64	4096	32	22	^15^
*A. baumannii*	MyNCGM 8	OXA-23, OXA-402 (OXA-51-like)	32	32	4	128	27	^15^
*A. baumannii*	MyNCGM 97	NDM-1, OXA-69	128	256	4096	64	25	^15^
*A. baumannii*	NCGM 237	OXA-23, OXA-66	16	16	4096	512	32	^15^
*A. baumannii*	IOMTU 442	OXA-70	0.25	2	4096	64	37	^21^
*A. baumannii*	IOMTU 449	OXA-98	1	2	4096	64	37	^21^
*A. baumannii*	NCGM 239	OXA-82 (OXA-51-like)	8	16	4096	32	31	^21^
*A. baumannii*	IOMTU 427	NDM-1	32	64	32	1	20	^22^
*A. baumannii*	IOMTU 430	NDM-1, OXA-23, PER-7	32	64	2048	128	22	^22^
*A. baumannii*	IOMTU 433	NDM-1, OXA-23, PER-7	64	128	4096	32	19	^22^
*A. baumannii*	IOMTU 436	OXA-23	16	64	4096	128	26	^22^
*A. baumannii*	IOMTU 437	NDM-1, OXA-23, PER-7	32	64	2048	256	21	^22^
*A. baumannii*	IOMTU 448	OXA- 23, PER-7	8	16	8	32	27	^22^
*A. baumannii*	IOMTU 450	NDM-1	64	128	4	64	19	^22^
*A. baumannii*	IOMTU 454	NDM-1, OXA-100	64	64	1024	32	20	^22^
*A. baumannii*	IOMTU 455	NDM-1, OXA- 23, PER-7	32	64	1024	128	22	^22^
*A. baumannii*	IOMTU 458	OXA-23, OXA-104	32	64	4096	128	23	^22^
*A. baumannii*	IOMTU 458	OXA- 23, PER-7	32	128	1024	1024	21	^22^
*A. baumannii*	IOMTU 460	OXA- 23, PER-7	32	128	2048	128	22	^22^
*A. baumannii*	IOMTU 461	OXA-23, OXA-371	8	16	32	32	29	^22^
*A. baumannii*	IOMTU 489	NDM-1, OXA-23	64	128	256	16	22	^22^
*A. baumannii*	IOMTU 496	OXA-23	8	16	32	32	27	^22^
*A. baumannii*	IOMTU 503	OXA- 23, PER-7	32	128	1024	1024	22	^22^
*A. baumannii*	IOMTU 508	PER-7	16	16	1024	64	27	^22^
*A. baumannii*	IOMTU 517	OXA- 23, PER-7	8	16	1024	32	27	^22^
*A. baumannii*	NCGM 241	OXA-66, OXA-72	128	128	4096	256	18	^22^
*A. baumannii*	NCGM 245	OXA-66, OXA-72	64	128	4096	256	21	^22^
*A. baumannii*	NCGM 253	OXA-66, OXA-72	64	256	4096	64	20	^22^
*A. baumannii*	NCGM 254	OXA-66, OXA-72	128	128	4096	32	19	^22^
*A. baumannii*	NCGM 257	OXA-66, OXA-72	64	64	4096	32	18	^22^
*A. baumannii*	MyNCGM 154	NDM-1, OXA-51-like	64	64	512	32	21	^24^
*A. baumannii*	MyNCGM 157_1	OXA-23, OXA-69	32	32	4096	32	24	^24^
*A. baumannii*	NCGM 169	OXA-23, TEM-1	64	8	1024	1024	29	^25^
*A. baumannii*	NCGM 170	OXA-23, TEM-1	64	32	2048	1024	27	^25^
*A. baumannii*	NCGM 174	OXA-51-like	32	32	2048	256	29	^25^
*A. baumannii*	NCGM 175	PER-1	32	32	1024	1024	29	^25^
*A. baumannii*	NCGM 182	TEM-1	32	32	1024	64	26	^25^
*A. baumannii*	NCGM 184	PER-1	32	32	128	1024	26	^25^
*A. baumannii*	NCGM 185	PER-1	32	32	32	1024	27	^25^
*A. baumannii*	NCGM 191	PER-1	64	64	32	1024	25	^25^
*A. baumannii*	NCGM 193	TEM-1	64	64	2048	1024	25	^25^
*A. baumannii*	NCGM 197	TEM-1	64	128	1024	512	24	^25^
*A. baumannii*	NCGM 207	TEM-1	64	128	2048	256	23	^25^
*A. baumannii*	NCGM 209	TEM-1	16	32	2048	64	27	^25^
*A. baumannii*	NCGM 211	OXA-51-like	64	64	2048	64	24	^25^
*A. baumannii*	NCGM 304	OXA-23, PER-1	16	16	32	1024	29	^25^
*A. baumannii*	NCGM 305	OXA-23	8	8	0.5	128	28	^25^
*A. baumannii*	NCGM 306	OXA-23, TEM-1	8	8	2048	64	28	^25^
*A. baumannii*	NCGM 308	OXA-23, TEM-1	16	64	1024	32	25	^25^
*A. baumannii*	NCGM 319	OXA-23, TEM-1	16	8	2048	1024	28	^25^
*A. baumannii*	NCGM 320	OXA-23, TEM-1	32	32	2048	512	24	^25^
*A. baumannii*	NCGM 321	OXA-23, TEM-1	128	64	2048	512	23	^25^
*A. baumannii*	NCGM 328	OXA-23	64	64	64	32	24	^25^
*A. baumannii*	NCGM 336	OXA-23, TEM-1	32	16	1024	1024	28	^25^
*A. baumannii*	NCGM 337	OXA-23, TEM-1	8	4	1024	256	34	^25^
*A. baumannii*	NCGM 340	OXA-23, TEM-1	32	32	1024	512	25	^25^
*A. baumannii*	NCGM 341	OXA-23, TEM-1	32	32	1024	512	25	^25^
*A. baumannii*	NCGM 346	OXA-23, TEM-1	32	32	1024	512	25	^25^
*A. baumannii*	NCGM 348	OXA-23	32	32	32	512	25	^25^
*A. baumannii*	NCGM 349	OXA-23, TEM-1	32	32	2048	256	25	^25^
*A. baumannii*	MyNCGM 123	OXA-23, PER-8, ADC	16	16	64	64	28	^26^
*A. baumannii*	MyNCGM 159	NDM-1, OXA-58, VEB-21, ADC	128	256	512	16	18	^26^
*A. baumannii*	MyNCGM 195	OXA-23, OXA-58, VEB-21, ADC	8	8	256	32	28	^26^
*A. baumannii*	MyNCGM 215	OXA-23, PER-7, ADC	16	16	2048	256	28	^26^
*A. baumannii*	MyNCGM 297	OXA-23, PER-7, ADC	16	64	2048	128	26	^26^
*A. baumannii*	MyNCGM 299	OXA-23, TEM-1D, ADC	64	64	4096	32	24	^26^
*A. baumannii*	MyNCGM 516	OXA-23, TEM-1D, ADC	64	64	4096	128	26	^26^
*A. baumannii*	MyNCGM 549	OXA-23, TEM-1D, ADC	128	128	4096	128	24	^26^
*A. baumannii*	MyNCGM 568	NDM-1, OXA-58, VEB-1, ADC	256	256	64	512	23	^26^
*A. baumannii*	MyNCGM 584	OXA-23, TEM-1D, ADC	64	128	2048	64	23	^26^
*A. baumannii*	MyNCGM 638	OXA-23, TEM-1D, ADC	128	128	4096	64	24	^26^
*A. baumannii*	MyNCGM 657	OXA-23, TEM-1D, ADC	128	128	4096	128	23	^26^
*A. baumannii*	MyNCGM 802	OXA-23, TEM-1D, ADC	16	16	2048	32	28	^26^
*A. baumannii*	MyNCGM 97	NDM-1, TEM-1D, ADC	128	256	2048	64	19	^26^
*A. bereziniae*	IOMTU 426	NDM-1	8	32	2	16	25	^15^
*A. indicus*	IOMTU 236	NDM-1	32	32	4096	4	26	^15^
*A. nosocomialis*	MyNCGM 45	OXA-23	32	32	4096	128	21	^15^
*A. nosocomialis*	MyNCGM 56	IMP-14	128	128	128	16	19	^15^
*A. nosocomialis*	MyNCGM 94_1	OXA-23	16	32	4096	2048	23	^15^
*A. pittii*	MyNCGM 427_3	OXA-23	32	32	4096	256	25	^15^
*A. radioresistens*	MyNCGM 223	OXA-23-like	0.25	0.25	1	1	44	^15^
*A. baumannii*	JU037	–	0.5	0.5	8	0.5	40	This study
*A. baumannii*	JU038	–	0.5	0.5	4	0.25	41	This study
*A. baumannii*	JU039	–	1	0.5	8	0.5	40	This study
*A. baumannii*	JU040	–	1	1	16	4	44	This study
*A. baumannii*	JU041	–	2	1	8	0.25	43	This study
*A. baumannii*	JU042	–	1	1	8	0.25	41	This study
*A. baumannii*	JU043	–	1	2	8	0.25	37	This study
*A. baumannii*	JU044	–	1	1	4	4	42	This study
*A. baumannii*	JU045	–	1	2	8	0.5	38	This study
*A. baumannii*	JU046	–	1	1	8	0.25	42	This study
*A. baumannii*	JU047	–	0.5	2	8	0.25	38	This study
*A. baumannii*	JU048	–	1	1	4	0.5	42	This study
*A. baumannii*	JU049	–	0.5	1	4	0.5	44	This study
*A. baumannii*	JU050	–	2	1	4	0.5	43	This study
*A. baumannii*	JU051	–	1	1	16	1	42	This study
*A. baumannii*	JU052	–	0.5	1	8	0.25	44	This study
*A. baumannii*	JU053	–	1	2	16	1	36	This study
*A. baumannii*	JU054	–	0.5	0.5	8	1	44	This study
*A. baumannii*	JU055	–	1	1	8	0.5	42	This study
*A. baumannii*	JU056	–	0.5	0.5	4	1	45	This study

^a^The isolate harbored an intrinsic *bla*_OXA-51-like_ or *bla*_ADC_ gene flanked by ISAba1 which is responsible for the production of an intrinsic charmapenemase, OXA-51-like β-lactamase, and an intrinsic cephalosporinase, ADC (AmpC) β-lactamase.

### Antimicrobial disk susceptibility tests

Antimicrobial susceptibility tests were performed using disks containing standard (10 μg), moderate (100 μg) and high (1000 μg) doses of meropenem. Each isolate was suspended in sterile saline and adjusted to a turbidity of 0.5 McFarland units. Isolates were inoculated onto Mueller–Hinton (MH) agar plates with sterile cotton swabs. A disk containing meropenem was placed onto the center of each plate, and the plates were incubated at 35 °C for 18 h to test *Pseudomonas* species or 24 h to test *Acinetobacter* species. The diameter (mm) of each inhibition zone was measured.

### Double disk synergy tests

Double disk synergy tests were performed using disks containing 1000 μg meropenem, together with disks containing the ESBL inhibitor clavulanic acid (40, 80 or 120 μg)^27^, or disks containing the MBL inhibitor sodium mercaptoacetate (3000, 6000 or 12,000 μg)^28^. Bacterial isolates were inoculated onto MH agar plates, and two disks, one containing meropenem and the other containing clavulanic acid or sodium mercaptoacetate, were separately placed on these plates at center-to-center distances of 5, 10 or 20 mm. The plates were incubated at 35 °C for 18 or 24 h, as described above, and the shape of each meropenem inhibition zone was determined.

### Microdilution method

The MICs of imipenem, meropenem, ciprofloxacin and amikacin were also determined by a microdilution method, as described by Clinical Standards Laboratory Institute guidelines^7^. Carbapenemase production was detected with the CIMTrisII carbapenem inactivation method (Kohjin Bio, Saitama, Japan)^24^.

### Ethical approval and consent to participate

This study was approved by the Biosafety Committee, Juntendo University [Approval Number BSL2/29-1].

## Results

### Antimicrobial disk susceptibility tests

**Figure 1:**
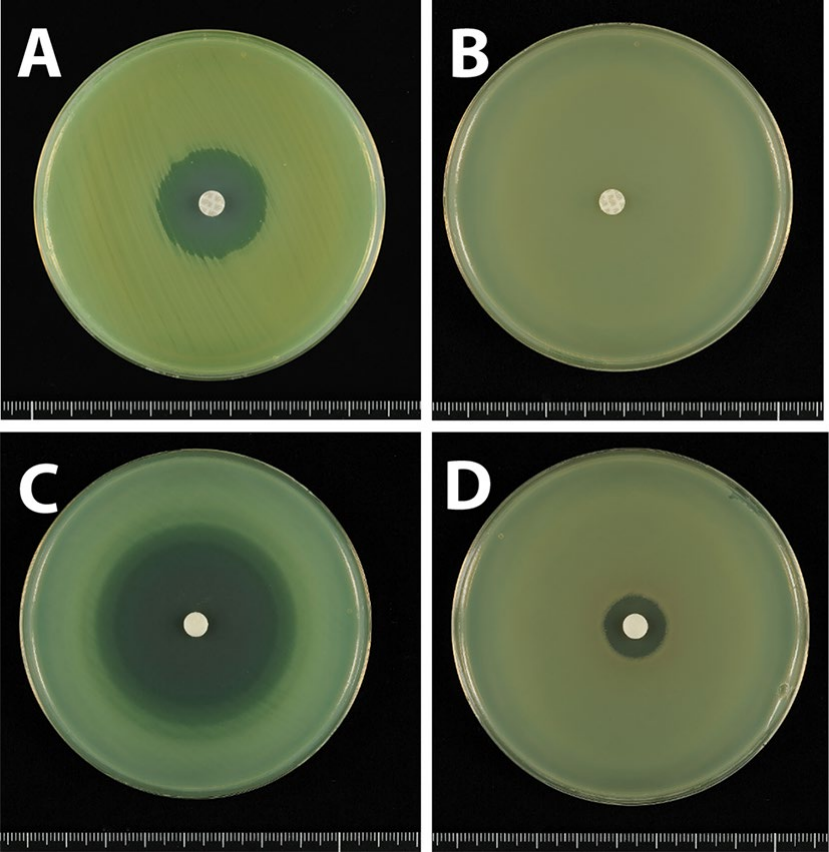
Antimicrobial susceptibility tests of (**A**,**C**) carbapenem-susceptible *P. aeruginosa* PAO1 (**B**,**D**) carbapenem-resistant *P. aeruginosa* IMCJ2.S1 on MH agar plates using disks containing (**A**,**B**) 10 μg or (**C**,**D**) 1000 μg meropenem.

Testing of antimicrobial susceptibility using standard disks containing 10 μg meropenem showed that carbapenem-susceptible *P. aeruginosa* PAO1 formed an inhibition zone of diameter 24 mm (Fig. 1A), whereas carbapenem-resistant *P. aeruginosa* IMCJ2.S1 did not form any inhibition zone (Fig. 1B). When tested with disks containing a moderate dose of meropenem (100 μg), PAO1 showed an inhibition zone of diameter 34 mm, whereas IMCJ2.S1 did not show any inhibition zone (date not shown). Using disks containing a high dose of meropenem (1000 μg), PAO1 and IMCJ2.S1 formed inhibition zones of diameter 50 mm (Fig. 1C) and 15 mm (Fig. 1D), respectively.

**Figure 2:**
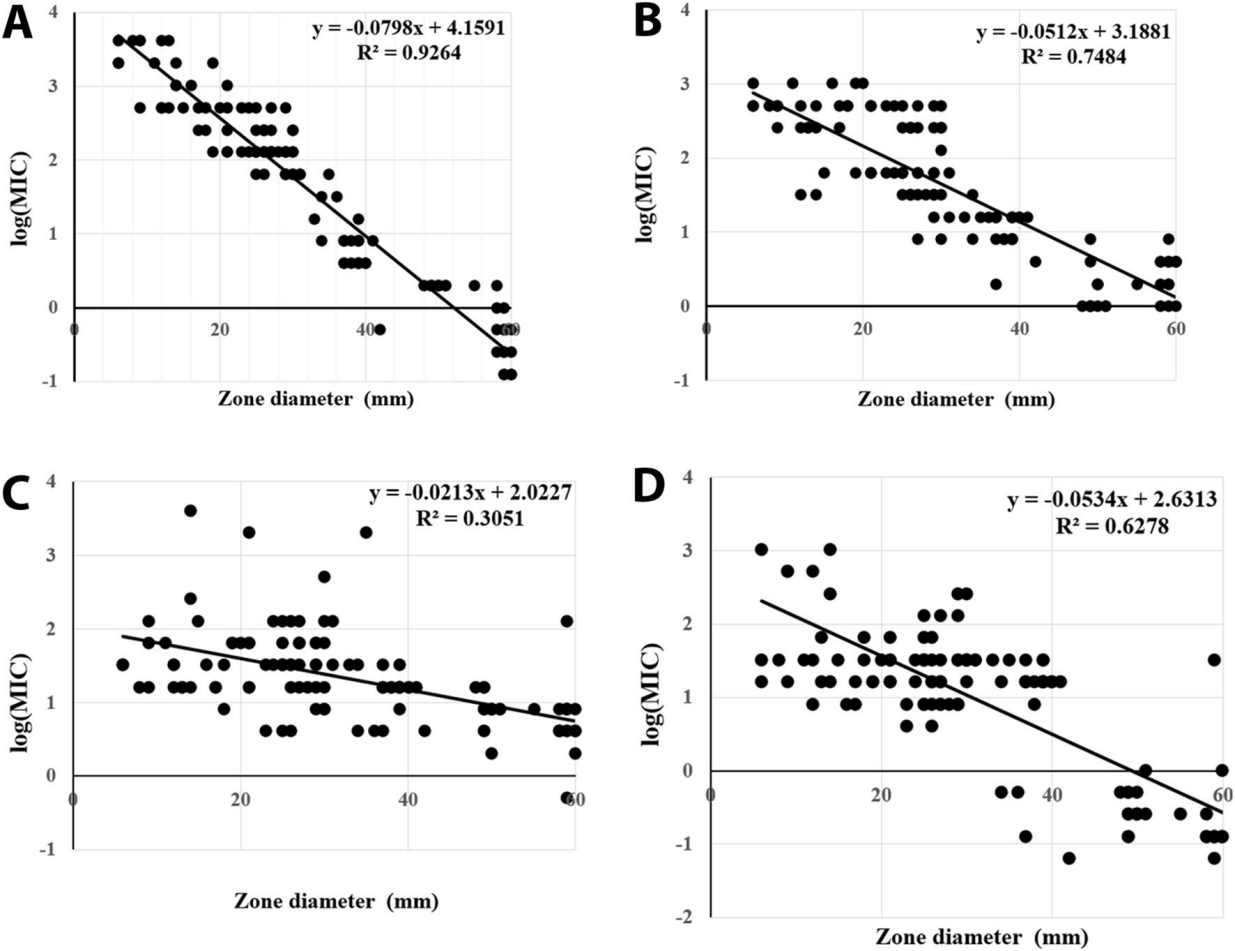
Correlations between inhibition zone diameters and MICs for (**A**) meropenem, (**B**) imipenem, (**C**) amikacin and (**D**) ciprofloxacin of *Pseudomonas* species.

**Figure 3:**
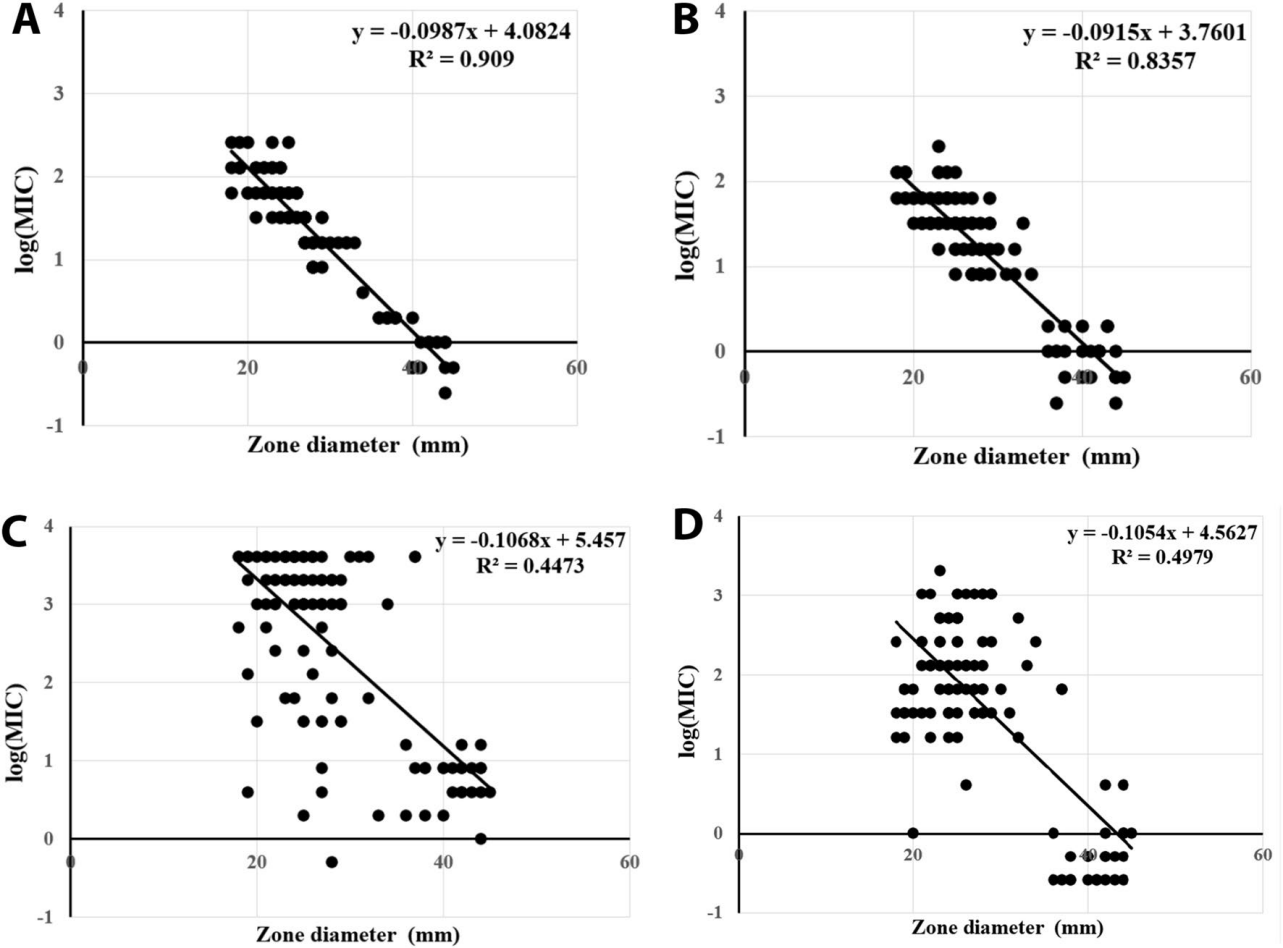
Correlations between inhibition zone diameters and MICs for (**A**) meropenem, (**B**) imipenem, (**C**) amikacin and (**D**) ciprofloxacin of *Acinetobacter* species.

Testing of *Pseudomonas* species showed that the diameters of inhibition zones were significantly negatively correlated with MICs for both meropenem (R^2^: 0.93) and imipenem (R^2^: 0.75) but did not correlate with MICs for amikacin (R^2^: 0.31) or ciprofloxacin (R^2^: 0.63) (Fig. 2). The diameters of inhibition zones of *Acinetobacter* species were also significantly negatively correlated with MICs for meropenem (R^2^: 0.91) and imipenem (R^2^: 0.84) but did not correlate with MICs for amikacin (R^2^: 0.45) or ciprofloxacin (R^2^: 0.50) (Fig. 3).

### Double disk synergy tests

**Figure 4:**
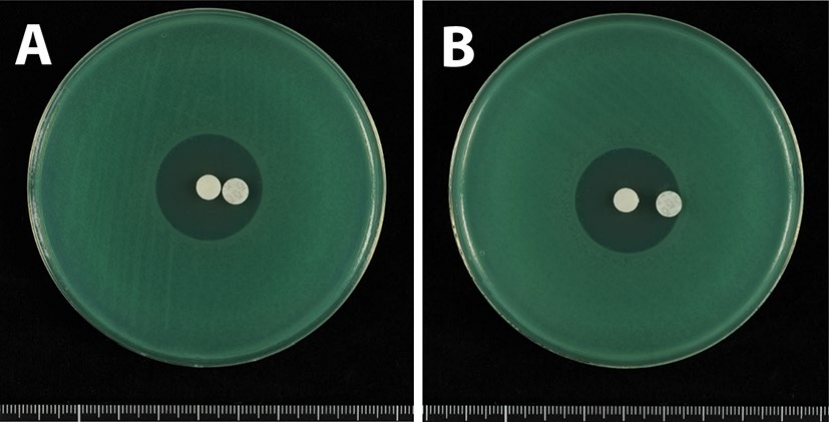
Double disk synergy tests of a *P. aeruginosa* isolate producing GES-5 using disks containing 1000 μg meropenem placed (**A**) 5 mm and (**B**) 10 mm from disks containing 10 μg clavulanic acid.

**Figure 5:**
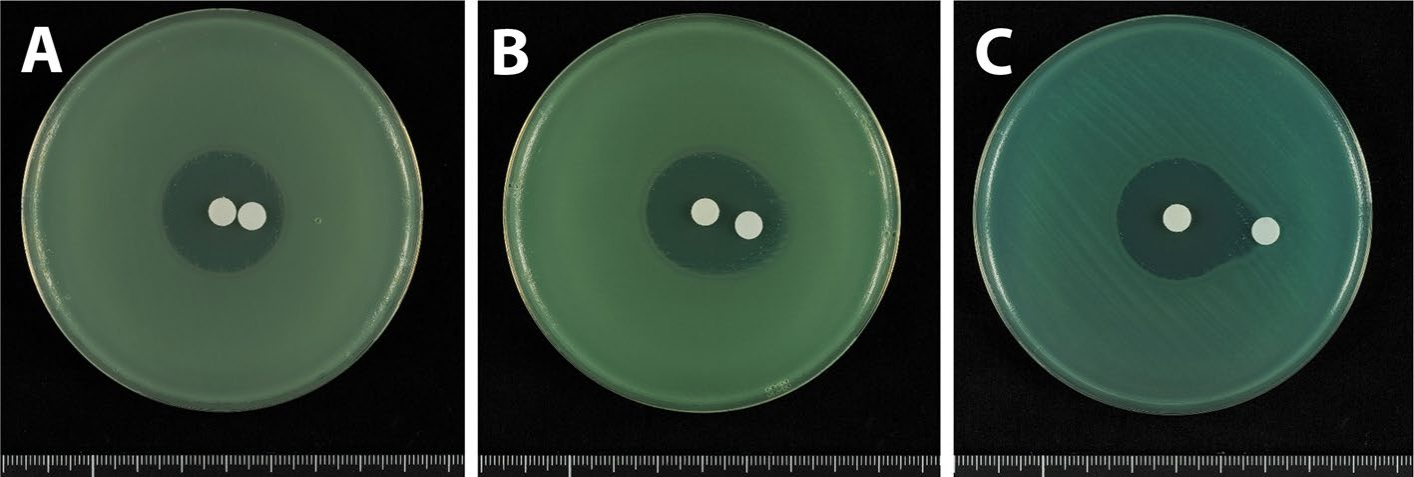
Double disk synergy tests of a *P. aeruginosa* isolate producing GES-5 using disks containing 1000 μg meropenem placed (**A**) 5 mm, (**B**) 10 mm and (**C**) 20 mm from disks containing 40 μg clavulanic acid.

**Figure 6:**
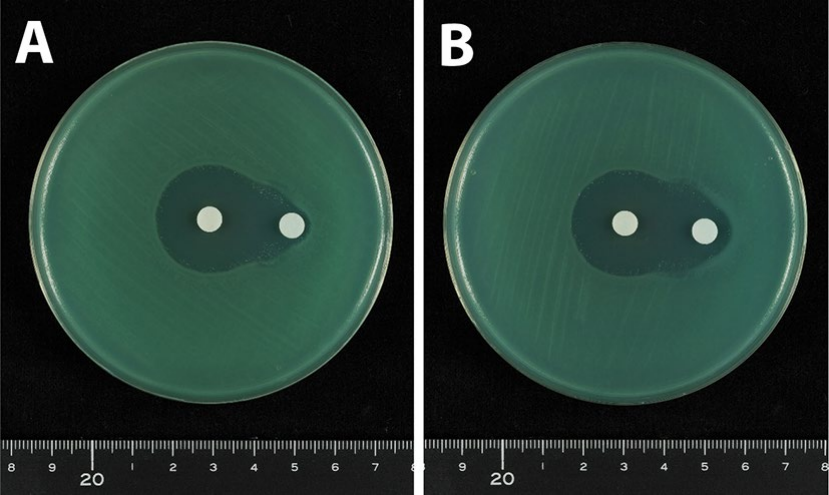
Double disk synergy tests of a *P. aeruginosa* isolate producing GES-5 using disks containing 1000 μg meropenem placed 20 mm from disks containing (**A**) 80 μg and (**B**) 120 μg clavulanic acid.

**Figure 7:**
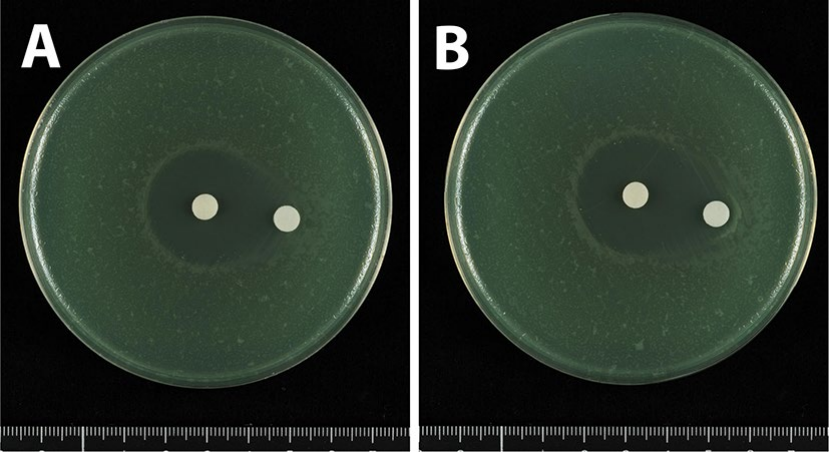
Double disk synergy tests of a *P. aeruginosa* isolate producing GES-24 using disks containing 1000 μg meropenem placed 20 mm from disks containing (**A**) 80 μg and (**B**) 120 μg clavulanic acid.

Double disk synergy tests were performed using a *P. aeruginosa* isolate that produced GES-5. Placement of a disk containing 10 μg clavulanic acid, together with a disk containing 1000 μg meropenem, on an MH agar plate at a distance of 5 or 10 mm showed that clavulanic acid did not alter the shape of the meropenem inhibition zone (Fig. 4). The shape of the meropenem inhibition zone was also unaffected by placement of a disk containing 40 μg clavulanic acid at distances of 5 and 10 mm (Fig. 5A,B). At a distance of 20 mm, the meropenem inhibition zone was slightly enhanced at the front of the disk containing 40 μg clavulanic acid, altering the shape of the meropenem inhibition zone (Fig. 5C). Placement of a disk containing 80 μg clavulanic acid 20 mm from the disk containing 1000 μg meropenem enhanced the meropenem inhibition zone at the front of the clavulanic acid-containing disk (Fig. 6A). This enhancement of the inhibition zone was also observed when a disk containing 120 μg clavulanic acid was placed 20 mm from the disk containing 1000 μg meropenem, although the clavulanic acid disk alone formed an inhibition zone (Fig. 6B). These findings showed that double disk synergy tests of a *P. aeruginosa* isolate producing GES-24 with clavulanic acid doses of 80 and 120 μg similarly enhanced the inhibition zone formed by 1000 μg meropenem (Fig. 7).

**Figure 8:**
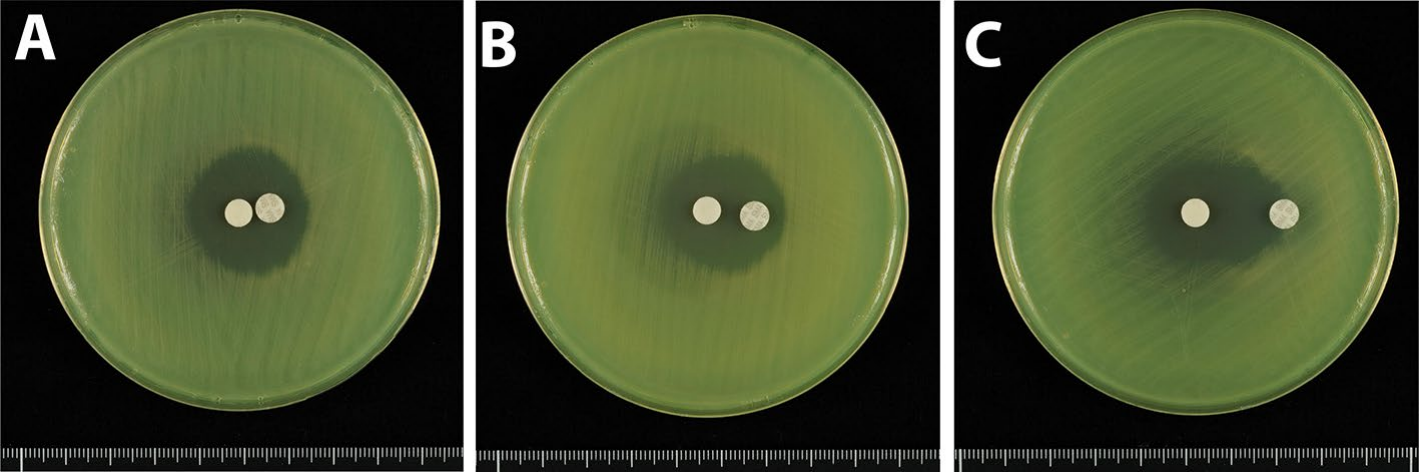
Double disk synergy tests of a *P. aeruginosa* isolate producing NDM-1 using disks containing 1000 μg meropenem placed (**A**) 5 mm, (**B**) 10 mm and (**C**) 20 mm from disks containing 3000 μg sodium mercaptoacetate.

**Figure 9:**
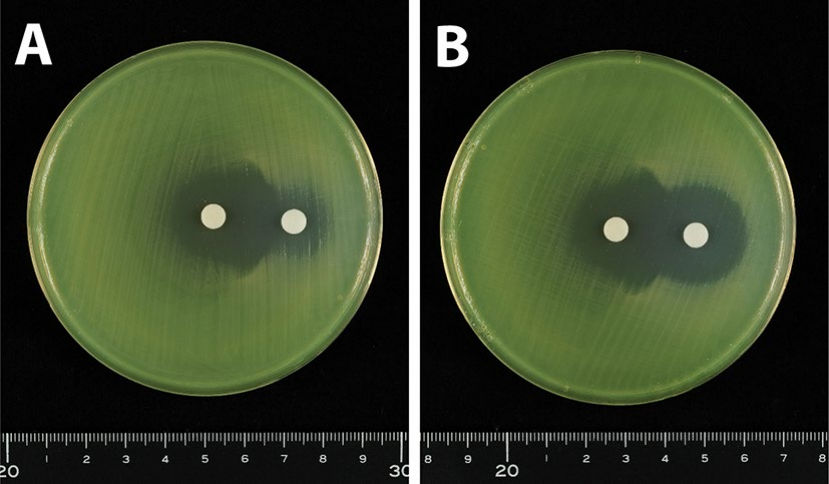
Double disk synergy tests of a *P. aeruginosa* isolate producing NDM-1 using disks containing 1000 μg meropenem placed 20 mm from disks containing (**A**) 6000 μg and (**B**) 12,000 μg sodium mercaptoacetate.

Double disk synergy tests were also performed using an isolate of NDM-1 producing *P. aeruginosa*. Separate placement of disks containing 1000 μg meropenem and 3000 μg sodium mercaptoacetate on MH agar plates at distances of 5, 10 and 20 mm showed that, at distances of 5 and 10 mm, the meropenem inhibition zone was slightly enhanced at the front of the disks containing sodium mercaptoacetate (Fig. 8A,B), with greater enhancement observed when the disks were at a distance of 20 mm (Fig. 8C). This enhancement of the inhibition zone was also observed following placement of a disk containing 6000 μg sodium mercaptoacetate (Fig. 9A). Moreover, a disk containing 12,000 μg sodium mercaptoacetate alone formed an inhibition zone (Fig. 9B), as did a disk containing 10 μl of 0.5 M EDTA, an inhibitor of metallo-β-lactamase such as NDM-1 (data not shown).

## Discussion

The present study found that antimicrobial susceptibility tests using disks containing a high dose of meropenem were able to quantitatively determine the carbapenem resistance of *Acinetobacter* and *Pseudomonas* species (Figs. 2, 3). Of the 125 clinical isolates of *Pseudomonas* species tested, 70 were highly resistant to meropenem, defined as having MICs ≥ 64 μg/ml for meropenem (Table 1), with 69 of these 70 highly carbapenem-resistant isolates of *Pseudomonas* having inhibition zones of diameter < 32 mm (Table 3). Moreover, all 42 highly carbapenem-resistant isolates of *Acinetobacter* species had inhibition zones of diameter < 27 mm (Table 3).

**Table 3:** Correlations between MIC values and inhibition zone diameters in isolates of *Pseudomonas* and *Acinetobacter* species^a^.

		Numbers of isolates with MICs of	
		≧64 µg/ml	< 64 µg/ml
*Pseudomonas* species (n = 103)			
Numbers of isolates with inhibition zone diameter of	< 32 mm	78	0
	≧ 32 mm	1	60
*Acinetobacter* species (n = 92)			
Numbers of isolates with inhibition zone diameter of	< 27 mm	42	15
	≧ 27 mm	0	55

^a^MICs of meropenem (the cutoff value of MIC: 64 µg/ml) were compared with inhibition zone diameters in isolates of *Pseudomonas* species (the cutoff value: 32 mm) and *Acinetobacter* species (the cutoff value: 27 mm).

These findings suggest that, in double disk synergy tests, a disk containing 1000 μg meropenem and a disk containing 80 μg clavulanic acid should be placed 20 mm apart on an MH agar plate to detect bacteria that produce class A carbapenems, whereas a disk containing 1000 μg meropenem and a disk containing 6000 μg sodium mercaptoacetate should be placed 20 mm apart on an MH agar plate to detect bacteria that produce class B metallo-β-lactamases. Although double disk synergy tests were optimized to detect ESBL and MBL producers, these tests failed to detect OXA-23 and KPC-2 producers. Similar results were observed with double disk synergy tests using standard disks that could not detect producers of OXA-type and KPC-type carbapenemases. Carbapenem inactivation methods, such as CIMTrisII^24^ are useful in the detection of *Pseudomonas* spp. and *Acinetobacter* spp. isolates that produce all types of carbapenemases, including OXA-type and KPC-type enzymes. These disks may be useful in bacteriological laboratories because of their technical ease, stability, and relatively low cost. Antimicrobial susceptibility tests using disks containing a high dose of meropenem will be useful in bacteriological laboratories because of their technical ease, stability, and relatively low cost. Although screening agar containing carbapenems has been shown useful in detecting carbapenem-resistant isolates of *Enterobacteriaceae*^29–31^, screening agar has not been routinely used in bacteriological laboratories because carbapenems in agar are chemically instable, making laboratory storage difficult.

## Conclusion

Susceptibility tests using disks containing a high dose of meropenem (1000 μg) can easily detect highly carbapenem-resistant isolates of *Acinetobacter* and *Pseudomonas* species in a quantitative manner. Double disk synergy tests using the disk containing meropenem with a disk containing a high dose of clavulanic acid (80 μg) or sodium mercaptoacetate (6000 μg) can detect ESBL and MBL producers, respectively, although these tests failed to detect OXA-23 and KPC-2 producers.

## Data Availability

The datasets analyzed during the current study are available from the corresponding author on request.

## Abbreviations

ESBL: Extended-spectrum β-lactamase
MBL: Metallo-β-lactamase
MH: Mueller-Hinton
MIC: Minimal inhibitory concentration
